# Integrated electromagnetic–circuit digital twin modelling for constraint-consistent optimal operation in heterogeneous multi-receiver wireless power transfer systems

**DOI:** 10.1038/s41598-026-50781-y

**Published:** 2026-04-29

**Authors:** Joungha Lee, Seung Beop Lee

**Affiliations:** 1 Department of Physical AI, Jeonbuk National University, Jeonju, 54896 Republic of Korea; 2 School of International Engineering and Science, Jeonbuk National University, Jeonju, 54896 Republic of Korea; 3 Institute of Applied Statistic, Jeonbuk National University, Jeonju, 54896 Republic of Korea; 4 JBNU-Purdue Research Institute (JPRI), Jeonbuk National University, Jeonju, 54896 Republic of Korea

**Keywords:** Wireless power transfer (WPT), Digital twin modelling, Electromagnetic–circuit coupling, Constraint-consistent optimal operation, Feasible operating region, Multi-receiver systems, Energy science and technology, Engineering, Mathematics and computing

## Abstract

Heterogeneous multi-receiver wireless power transfer (WPT) systems exhibit strong electromagnetic coupling and non-uniform load requirements, making it difficult to determine an operating point that is simultaneously feasible for all receivers and efficient at the system level. This study presents an integrated electromagnetic–circuit digital twin modelling approach that enables constraint-consistent optimal operation in single-transmitter, multi-receiver WPT systems with mixed compensation topologies. A design-stage digital twin is constructed by tightly coupling three-dimensional electromagnetic-field simulation with equivalent circuit modelling. Self- and mutual-inductance parameters are extracted from the physical coil configuration and mapped into the circuit model, enabling physics-consistent evaluation of receiver delivered power and total transmission efficiency under interacting receiver conditions. The operating point is determined by enforcing receiver-specific rated power requirements as feasibility constraints while refining system efficiency within the feasible region. The approach is demonstrated on a 1T–3R WPT case study comprising two series-compensated receivers and one parallel-compensated receiver with non-uniform rated powers. The results show that the proposed digital twin modelling identifies an operating condition that satisfies all receiver power constraints while improving overall efficiency. A one-dimensional parameter sweep around the obtained operating point further confirms local robustness and sensitivity characteristics within the simulation-based design-stage framework, supporting the validity of the constraint-consistent optimum for heterogeneous multi-receiver configurations.

**Supplementary Information:**

The online version contains supplementary material available at 10.1038/s41598-026-50781-y.

## Introduction

Wireless power transfer (WPT) is no longer confined to device-level convenience but has evolved into an energy delivery technology spanning medical devices, consumer electronics, vehicles, charging infrastructure, and power systems. Recent reviews have also emphasized that magnetically coupled resonant WPT is expanding toward high-degree-of-freedom and multi-pickup charging scenarios in IoT-related applications, where spatial freedom, multi-device interaction, and system-level optimization become key design challenges^1^. In biomedical and miniature systems, wireless charging has been demonstrated as a viable alternative to physical connectors, enabling long-term operation of implantable devices while revealing that thermal safety and power delivery limitations must be addressed at the system level rather than at the component level^2,3^. Further studies on implantable and wearable wireless charging architectures indicate that spatial uncertainty arising from implantation depth, tissue variability, and relative positioning is an inherent characteristic of practical systems, directly affecting magnetic coupling and delivered energy^4^. In consumer electronics, free-positioning wireless charging systems based on three-dimensional transmitting coils illustrate a transition from fixed alignment toward user-driven spatial variability, thereby expanding the design problem from a single operating point to a system-level energy delivery space^5^,^6^. At transportation power levels, dual-receiver EV charging architectures demonstrate that WPT must support regulated charging behaviors, such as constant-current and constant-voltage characteristics, across interacting receivers rather than for a single isolated load^7^. Comprehensive reviews of inductive WPT for electric vehicles further emphasize that practical performance is jointly governed by magnetic coupler design, compensation topology, safety requirements, and operating scenarios, reinforcing the system-level nature of WPT technology^8^. Dynamic wireless charging surveys additionally highlight that electromagnetic, circuit, and control domains are inherently coupled in real deployments, confirming that WPT design cannot be treated as a single-domain problem^9^. Beyond individual vehicles, wireless charging is increasingly discussed as part of charging infrastructure and vehicular energy networks, where interoperability and deployment considerations extend beyond device-level optimization^10,11^. From a power-system perspective, recent analyses indicate that dynamic wireless charging can influence load profiles, motivating system-level modelling that captures interactions between operating conditions and energy delivery constraints^12^.

As WPT systems scale in power level, spatial coverage, and application scope, design constraints do not simply increase in number but interact in a nonlinear and often competing manner. Analyses of coupling variation in free-positioning and nonoverlapping charging environments show that abrupt changes in magnetic coupling can induce hazardous or unstable operating states, revealing that coupling dynamics act as a dominant system-level constraint rather than a minor perturbation^7^. In high-current AGV charging systems, experimental evidence further indicates that coupling-induced imbalance can persist under positional offset and load variation, directly constraining feasible operating regions^13^. Bidirectional AGV wireless charging architectures expand the operating envelope to multi-mode energy exchange, but simultaneously introduce additional constraints on power routing and allocation across modes^14^. Dynamic AGV charging studies based on communication-free optimal frequency control confirm that misalignment-induced efficiency degradation must be continuously mitigated during operation, underscoring the persistent nature of coupling variation^15^. Regulatory and safety requirements add another layer of constraints^16–18^. Environmental and structural factors further complicate system behavior^19^. At the architectural level, analytical investigations across SS, SP, PS, and PP compensation families clarify that topology selection directly governs reflected impedance behavior and load interaction, making topology heterogeneity a first-order constraint^20^. EV dynamic wireless charging coil optimization for constant-output behavior further demonstrates that geometry and parameter selection must be jointly considered under motion-induced coupling variation^21^. Repeater-coil-assisted interactive power transmitting approaches also confirm that accommodating misalignment often introduces additional resonant elements, which increase degrees of freedom and intensify constraint interactions^22^. Taken together, these studies demonstrate that as WPT systems expand, constraints arising from coupling dynamics, regulatory requirements, environmental effects, and topology heterogeneity do not act independently but interact nonlinearly, fundamentally shaping feasible design and operation.

Given the nonlinear interaction of constraints in scaled WPT systems, a wide range of solution strategies has been proposed; however, these efforts largely address isolated aspects of the problem. Control-oriented approaches focus on mitigating operational disturbances after they arise^23–25^, and communication-free optimal-frequency control for dynamic AGV charging similarly demonstrates that misalignment-induced efficiency degradation must be continuously compensated during operation^15^. Parallel efforts propose converter- and topology-level architectures to enhance controllability and integration^17,18,26,27^. At the coupler and layout level, physics-based optimization has been actively pursued^28–35^. Finally, feasibility and system-scale studies highlight both the promise and limitations of current approaches, showing that high-fidelity modelling can guide large-scale system design but also revealing substantial computational demands and integration challenges^36,37^. Taken together, these studies indicate that existing solutions address control, converter architecture, coil design, or optimization in isolation, while a unified design-stage approach that consistently couples electromagnetic behavior, circuit-level performance, and heterogeneous receiver requirements remains difficult to realize.

Despite the progress outlined above, what remains missing is an integrated design-stage framework that unifies electromagnetic behavior, circuit-level performance, and receiver-specific requirements for heterogeneous multi-receiver WPT systems. Recent studies on multi-receiver wireless charging have moved toward constraint-aware formulations by explicitly incorporating receiver-side voltage constraints to improve efficiency and feasibility^38^. Related optimization-based approaches have further demonstrated that different rated powers required by individual receivers can be treated as explicit constraints when determining optimal resonant conditions under altered coupling effects^39^. Transmitter-module optimization for single-transmitter–multiple-receiver systems also indicates that system-level performance can be improved by systematically tuning transmitter-side design variables rather than relying solely on receiver-side adjustments^40^. While these studies represent the closest prior art to the present work, they typically assume homogeneous receiver topologies, fixed electromagnetic coupling models, or limited separation between electromagnetic analysis and circuit evaluation, and therefore do not provide a physics-consistent digital-twin representation that tightly couples electromagnetic fields and circuit behavior within a unified design-stage loop for heterogeneous receiver configurations. Related studies have also demonstrated that accurate parameter extraction and physics-consistent equivalent circuit representation are essential for reliable WPT modelling and subsequent optimization or control, particularly when direct analytical characterization of complex electromagnetic structures is difficult^41^. However, such parameter-extraction-oriented approaches have not been developed for the heterogeneous multi-receiver operating-point determination problem considered in the present work.

To address this gap, this paper proposes integrated electromagnetic–circuit digital twin modelling to determine constraint-consistent optimal operation in heterogeneous multi-receiver WPT systems. The proposed digital twin couples electromagnetic field analysis with equivalent circuit evaluation, enabling system-level performance prediction under varied receiver configurations. Receiver-specific rated power requirements are enforced as feasibility constraints, and the operating condition is refined within the feasible region to obtain an optimal, constraint-consistent solution. The effectiveness of the proposed approach is demonstrated using a representative single-transmitter–three-receiver WPT system with mixed series and parallel compensation topologies, and local robustness is examined through a one-dimensional parameter sweep around the obtained operating point.

The remainder of this paper is organized as follows. Section 2 introduces the heterogeneous multi-receiver WPT model and the constraint-consistent problem formulation. Section 3 describes the construction of the digital twin and the operating point determination workflow. Section 4 presents the case study and performance evaluation results, and Sect. 5 concludes the paper with a discussion of limitations and future research directions.

## Method

**Fig. 1:**
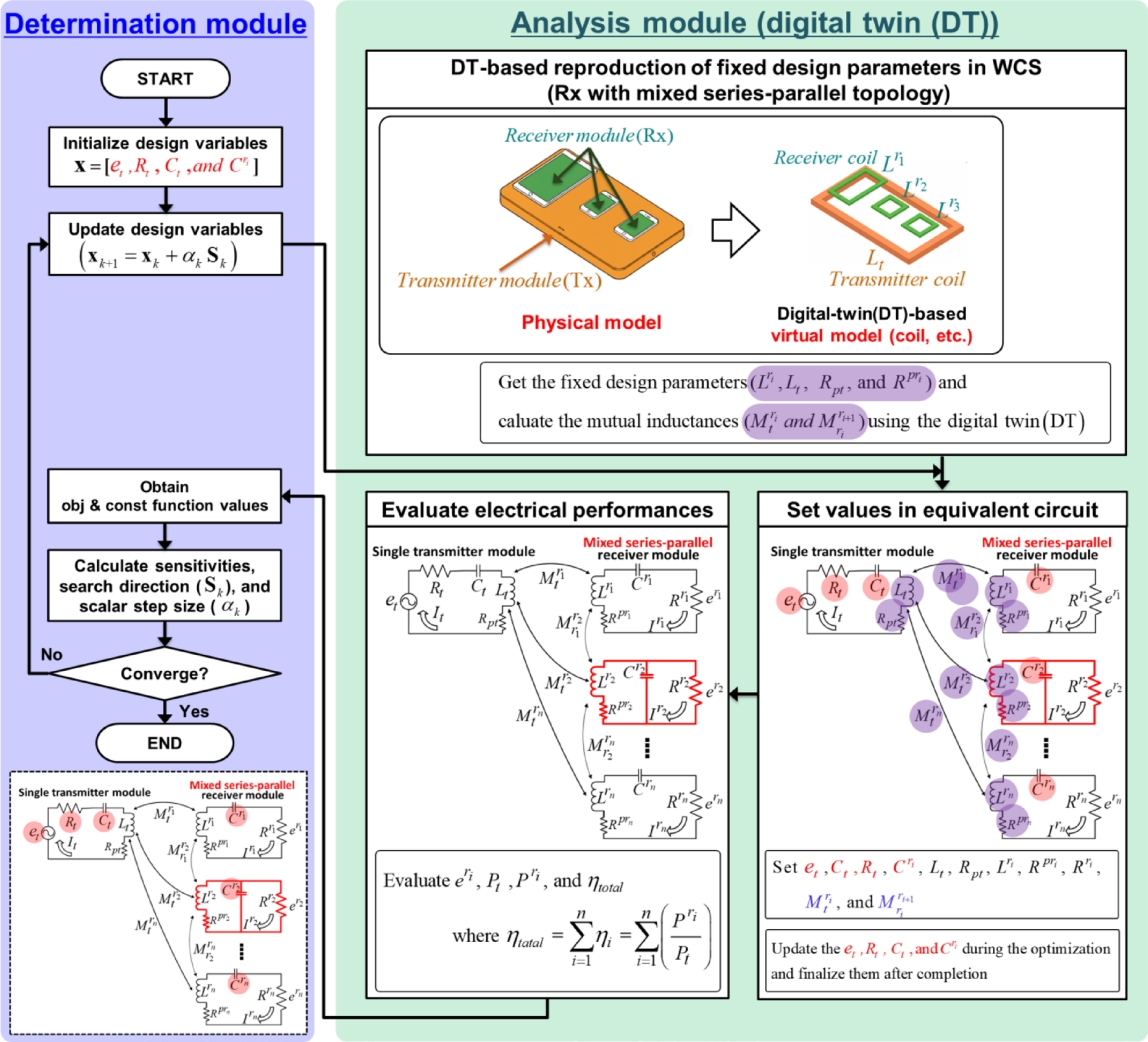
Conceptual illustration of the integrated electromagnetic–circuit digital twin workflow for constraint-consistent operating point determination in a multi-receiver WPT system with heterogeneous receivers. All figures in this study were created by the authors using simulation outputs, software-generated schematics, or original illustrations.

This section describes the integrated electromagnetic–circuit digital twin modelling workflow used to determine a constraint-consistent optimal operating condition for heterogeneous multi-receiver wireless power transfer (WPT) systems. Rather than treating optimization as the primary contribution, the methodology focuses on establishing a physics-consistent mapping between physical coil configurations, electromagnetic coupling characteristics, and circuit-level performance. The digital twin enables systematic evaluation of receiver-specific power constraints under interacting multi-receiver conditions and provides a reproducible procedure for selecting an operating point within the feasible region Fig. 1.

The workflow consists of two tightly coupled components: (i) a digital twin–based analysis module that links electromagnetic-field simulation with equivalent circuit modelling, and (ii) a numerical search procedure that iteratively examines operating conditions to identify a constraint-consistent optimum. The following subsections describe the construction of the digital twin, the formulation of the operating-point determination problem, and the iterative procedure used in this study.

### Analysis module (digital twin–based system evaluation)

The analysis module is constructed as a design-stage digital twin (DT) that provides a physics-consistent virtual replica of the target single-transmitter to multiple-receiver (S–M) WPT system. Unlike conventional sequential co-simulation, the proposed DT establishes a closed and repeatable mapping between the physical configuration, electromagnetic coupling characteristics, and circuit-level performance metrics, enabling systematic evaluation during the optimization process.

Given a fixed physical layout of the transmitter and receiver coils, the DT first reproduces the electromagnetic characteristics of the system. Self-inductance and resistance parameters of the transmitter coil and each receiver coil (e.g., *L*_*t*_, *L*^*ri*^, *R*^*ri*^ ​, and parasitic resistance terms (*R*^*pri*^)) are defined according to the physical geometry and material properties. These parameters are imported into a three-dimensional electromagnetic-field solver (ANSYS Maxwell), where the spatial arrangement of the coils is used to compute the mutual inductance terms between the transmitter and each receiver, as well as coupling interactions among receivers when applicable. The resulting mutual inductance matrix captures the coupling characteristics of the physical system under the given configuration and serves as a key interface between the physical domain and the circuit domain. In the present formulation, the electromagnetic parameters are extracted for a fixed spatial configuration of the transmitter and receiver coils, and the resulting mutual inductance matrix is treated as invariant during the operating-point determination process. Therefore, positional shift or misalignment, which alters the coupling coefficients, is not explicitly considered in the current model. Extending the framework to such scenarios requires treating the mutual inductance matrix as a position-dependent parameter set and updating it across different spatial configurations. The extracted electromagnetic parameters are then transferred to an equivalent-circuit model implemented in ANSYS Simplorer. In this model, each receiver is represented using its corresponding series or parallel compensation topology, allowing mixed series–parallel receiver configurations to be explicitly modeled within a unified circuit framework. Using the DT-based equivalent circuit, the electrical behavior of the S–M WPT system is evaluated, including the transmitter-side current and voltage, the power delivered to each receiver, and the total transmission efficiency. These performance metrics are computed under steady-state operation and are treated as deterministic outputs of the DT for the given design-variable set. In the present study, the receiver-side load is represented implicitly through fixed rated-power requirements under steady-state conditions. This assumption allows the operating-point determination problem to be formulated as a constraint-consistent design-stage problem under a fixed electromagnetic configuration. In practical battery-powered applications, however, the effective load varies across charging stages, such as constant-current and constant-voltage operation. Extending the present framework to such scenarios requires modeling receiver-side loads as time-varying parameters or as a set of representative charging states, and evaluating the digital twin across these conditions. This enables the identification of robust or state-dependent feasible operating conditions under variable-load environments.

The analysis module thus provides a consistent parameter-to-performance mapping that synchronizes electromagnetic-field evaluation and circuit-level analysis. The resulting performance metrics are passed to the optimization module as inputs for objective and constraint evaluation, enabling iterative design updates within the optimization loop. By maintaining a fixed and explicit interface between physical parameters, electromagnetic coupling, and circuit performance, the DT-based analysis module ensures that design decisions are evaluated in a reproducible and physics-consistent manner throughout the optimization process.

### Operating-point determination module

The operating-point determination problem is formulated as a constrained nonlinear search in which receiver-specific rated power requirements are explicitly enforced as feasibility constraints. Rather than treating efficiency maximization as an isolated objective, the procedure seeks an operating condition that satisfies all receiver constraints while achieving high system-level transmission efficiency.

Let the design-variable vector be defined as ${\mathrm{x}}=[e_{t}^{{}},\,R_{t}^{{}},\,\,C_{t}^{{}},\,\,\,C_{{}}^{{{r_i}}},...]\,\,\,\,\,(i=1,\,2,\,3,...,n)$ consisting of transmitter-side excitation parameters and compensation-related elements that can be practically adjusted during the design stage and significantly influence power transfer characteristics. The objective is to identify an operating condition that maximizes overall transmission efficiency of the S–M WPT system subject to inequality constraints ensuring that each receiver meets its rated power requirement within a prescribed tolerance. This formulation directly reflects the system-level requirement that heterogeneous receivers must be simultaneously supported under a single transmitter.

The constrained search is implemented using a deterministic gradient-based numerical routine. In this study, the Modified Method of Feasible Direction (MMFD)^37^ is employed as a computational tool to update design variables while maintaining feasibility. The algorithm determines search directions based on local sensitivity information of the objective and constraint functions, ensuring that candidate operating points remain within the feasible region.

At each iteration, the current design-variable set is evaluated through the DT-based analysis module described in Sect. 2.1, which returns the corresponding objective and constraint values. The numerical routine then updates the variables accordingly. This tightly coupled electromagnetic–circuit evaluation loop enables physics-consistent identification and refinement of a constraint-consistent optimal operating condition without reliance on surrogate models or empirical tuning.

### Iterative procedure

The DT evaluation provides the power delivered to each receiver and the total transmission efficiency under steady-state conditions. These quantities are used to assess feasibility with respect to receiver-specific power constraints and to guide refinement within the feasible region. Local sensitivity information is estimated numerically to determine search directions that preserve constraint satisfaction while improving performance.

Based on the computed sensitivities, a feasible update direction **S**_*k*_ is identified, and the design variables are updated as

$$x_{{k + 1}} = x_{k} + \alpha _{k} S_{k}$$

where *α*_*k*_ denotes the step size along the feasible direction. The update is performed such that the new operating point remains within the feasible design space defined by receiver-level power constraints.

The iterative process first seeks a region in which all receiver constraints are satisfied. Once feasibility is achieved, subsequent iterations refine the operating condition within the feasible region to improve system-level efficiency. Convergence is declared when changes in the objective function fall below a prescribed threshold and all receiver constraints remain satisfied within tolerance. Through this DT-coupled iterative procedure, a constraint-consistent optimal operating condition is systematically identified for heterogeneous multi-receiver configurations.

### Computational settings and cost

All simulations were performed on a workstation equipped with an Intel^®^ Core™ i5-10400–class CPU operating at 2.90 GHz and 32 GB RAM under a Windows 10 environment. Electromagnetic-field analysis was conducted using ANSYS Maxwell, while circuit-level evaluation was carried out in ANSYS Simplorer. The two environments were coupled through in-house scripts that transferred extracted electromagnetic parameters to the circuit model.

Within the proposed digital twin modelling approach, electromagnetic-field simulation primarily serves as a one-time parameter extraction step. Once the physical geometry and relative positions of the transmitter and receiver coils are fixed, the self- and mutual-inductance parameters remain invariant. This assumption holds only for a fixed spatial configuration. When positional variation or misalignment is introduced, the electromagnetic parameters no longer remain invariant, and repeated parameter extraction or update mechanisms are required to capture position-dependent coupling effects. Consequently, the electromagnetic simulation is performed only once to establish the digital twin parameter set, and subsequent operating-point determination relies mainly on repeated circuit-level evaluations. At each iteration, the circuit model is updated with revised design variables, and receiver delivered power and total transmission efficiency are computed under steady-state conditions. Sensitivity information is estimated numerically to guide the search within the feasible region. The computational cost therefore scales primarily with the number of design variables and circuit evaluations, rather than with repeated full electromagnetic simulations. Because the approach separates one-time electromagnetic parameter extraction from iterative circuit evaluation, the overall computational burden remains tractable and suitable for offline design-stage analysis. This balance between electromagnetic fidelity and computational cost enables practical modelling of heterogeneous multi-receiver WPT systems without reliance on surrogate approximations.

## Application: case study and results

This section presents a representative case study to demonstrate constraint-consistent operating point determination in a practical one-to-many wireless power transfer (WPT) scenario with heterogeneous receivers. A single-transmitter, three-receiver (1T–3R) system is considered, where the receivers employ mixed series and parallel compensation topologies and have non-uniform rated power requirements. The case study first reports the constraint-consistent optimal operating condition obtained from the proposed digital twin modelling workflow and then evaluates its consistency and local robustness through a one-dimensional (1D) parameter sweep around the obtained operating point under identical simulation conditions. The following subsections describe the system configuration, operating-point determination setup, convergence behavior, and resulting performance.

### Case study description and system configuration

**Fig. 2:**
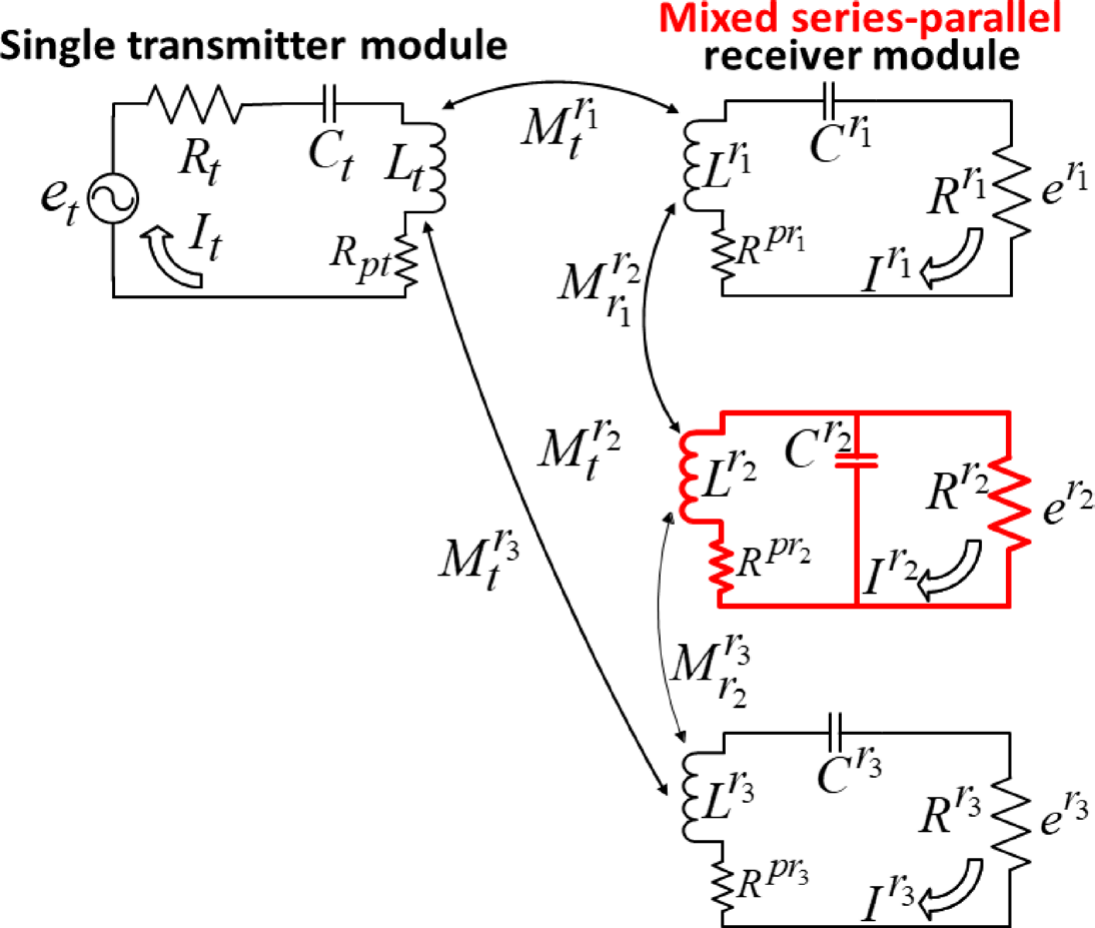
Circuit representation of the single-transmitter, three-receiver (1T–3R) WPT system with heterogeneous receiver compensation topologies (two series-compensated receivers and one parallel-compensated receiver.

The case study considers a single-transmitter to three-receiver (1T–3R) wireless power transfer (WPT) system designed to represent a practical heterogeneous charging scenario. The transmitter is driven by a single power source and operates at a fixed excitation frequency of 400 kHz, while the three receivers are simultaneously supplied through magnetic coupling. As illustrated in Fig. 2, the target WPT system consists of one transmitter module and three receiver modules, forming a representative one-to-many energy delivery configuration.

To reflect realistic multi-load conditions, the receiver modules employ mixed compensation topologies and non-uniform rated power requirements. Receiver 1 ($r_{{\,1}}^{{}}$) adopts a series-compensated topology with a rated power of 12.5 W, representing a relatively higher-power charging demand within the system. Receivers 2 ($r_{{\,2}}^{{}}$) and 3 ($r_{{\,3}}^{{}}$) are rated at 5 W each, corresponding to lower-power charging demands. To introduce circuit-level heterogeneity independent of geometric effects, Receiver 2 employs a parallel compensation topology, whereas Receiver 3 adopts a series compensation topology, while both receivers utilize identical coil geometries and dimensions. This configuration enables isolation of the effects of compensation topology on power regulation and system-level optimization.

**Fig. 3:**
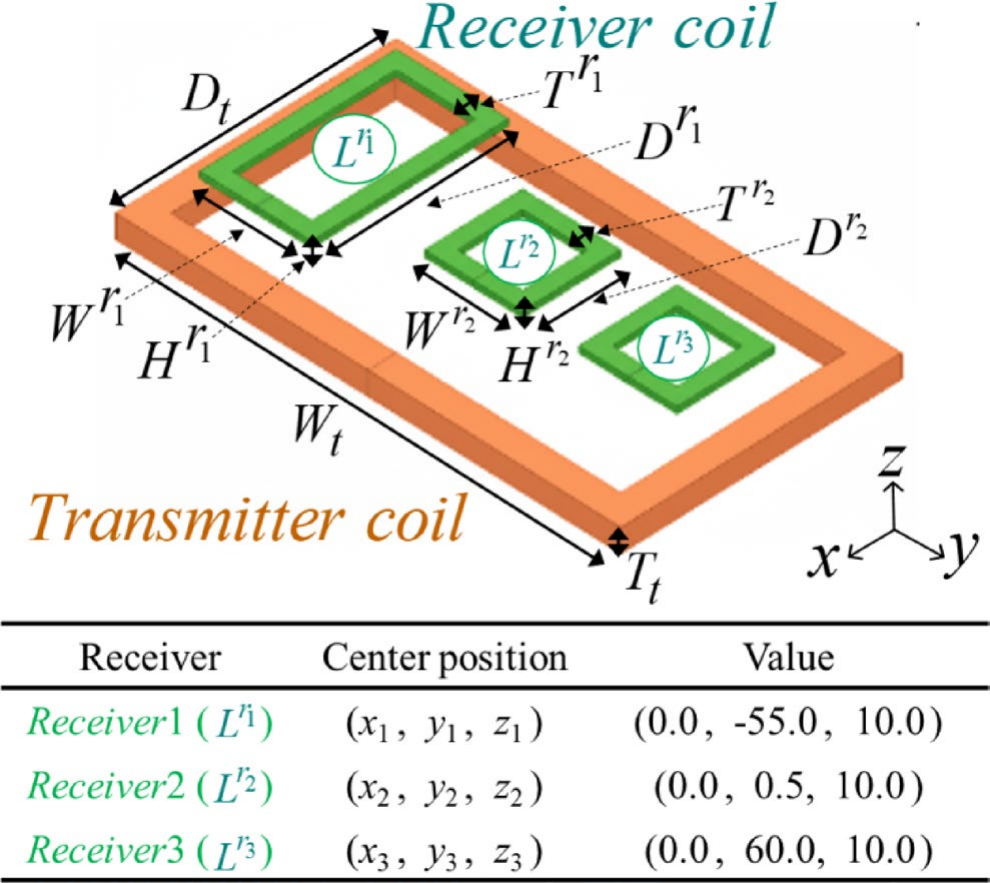
Physical layout of the transmitter and receiver coils in the single-transmitter, three-receiver (1T–3R) WPT system, showing the spatial configuration used for electromagnetic parameter extraction in the digital twin framework.

All receiver modules are positioned at a fixed air gap of 10 mm above the transmitter coil, and the physical layout of the module configuration is depicted in Fig. 3. The geometric arrangement, including the relative positions and orientations of the transmitter and receiver coils, is held constant throughout the case study. Under this assumption, electromagnetic coupling characteristics—expressed through self- and mutual-inductance parameters—are extracted once using the DT-based analysis module. These physical and circuit parameters are imported into the digital twin framework and implemented in ANSYS Maxwell, and the resulting mutual inductance values among all coils are summarized in Table 2. Detailed coil and circuit specifications of each module are provided in Table 1; Fig. 2.

Each receiver’s rated power is treated as a fixed system specification rather than an adjustable design variable and is enforced as an explicit constraint during the optimization process. This formulation assumes steady-state operating conditions for each receiver and does not explicitly model time-varying load behavior associated with practical battery charging processes. The fixed rated-power specification is adopted here to isolate the constraint-consistent operating-point determination problem under heterogeneous receiver requirements. The resulting non-uniform power distribution and mixed compensation topologies create an inherently asymmetric optimization problem, in which satisfying receiver-specific power requirements cannot be achieved through uniform or tuning-based approaches. Instead, coordinated adjustment of transmitter-side excitation and compensation-related design variables is required to simultaneously meet heterogeneous receiver demands under a single transmitter.

In this case study, the design variables are therefore limited to transmitter-side excitation and compensation-related parameters that can be adjusted at the design stage without altering the physical layout. All other parameters—including coil geometries, material properties, relative positioning, and operating frequency—are kept fixed to ensure that the optimization focuses on system-level energy delivery and efficiency improvement under heterogeneous receiver requirements, rather than on geometric redesign. This setup provides a clear and controlled basis for evaluating how the proposed DT-based optimization framework resolves competing objectives of receiver-specific power constraint satisfaction and overall system efficiency maximization.

**Table 1:** Physical and electrical parameters of the single-transmitter, three-receiver (1T–3R) WPT system used in the case study.

Design constants	Transmitter module	Receiver module	
		${r_{\,1}}$ (12.5 W)	${r_{\,2}}\,\,\,{\mathrm{and}}\,\,\,{r_{\,3}}\,$ (5 W)
Length (mm)	$D_{t}^{{}}$= 100.00	$D_{{}}^{{{r_1}}}$= 70.00	$D_{{}}^{{{r_2}}}$= 35.00
Width (mm)	$W_{t}^{{}}$= 180.00	$W_{{}}^{{{r_1}}}$= 40.00	$W_{{}}^{{{r_2}}}$= 35.00
Height (mm)	$H_{t}^{{}}$= 7.50	$H_{{}}^{{{r_1}}}$= 2.00	$H_{{}}^{{{r_2}}}$= 2.00
Thickness (mm)	$T_{t}^{{}}$= 10.00	$T_{{}}^{{{r_1}}}$= 6.00	$T_{{}}^{{{r_2}}}$= 6.00
Coil turns	$N_{t}^{{}}$= 30.00	$N_{{}}^{{{r_1}}}$= 24.00	$N_{{}}^{{{r_2}}}$= 24.00
Self-inductance ($\mu {\mathrm{H}}$)	$L_{t}^{{}}$= 248.45	$L_{{}}^{{{r_1}}}$= 55.95	$L_{{}}^{{{r_2}}}$= 27.55
Resistance (Ω)	$R_{t}^{{}}$= 1	$R_{{}}^{{{r_1}}}$= 10	$R_{{}}^{{{r_2}}}$= 5

### Design variables, objective, and constraints

This subsection describes the formulation used to determine the constraint-consistent operating condition for the 1T–3R case study, including the selected operating variables, the performance metric, and the receiver-specific feasibility constraints. Within the proposed design-stage digital twin modelling approach, electromagnetic parameters associated with the fixed coil geometry and spatial configuration (e.g., self- and mutual-inductance terms summarized in Tables 1 and 2) are treated as constants. In contrast, circuit-level and excitation-related parameters that can be practically adjusted without altering the physical layout are treated as operating variables.

**Table 2:** Mutual inductance values of the transmitter–receiver and receiver–receiver coil pairs in the 1T–3R WPT case study.

Category	$M_{t}^{{{r_1}}}$($\mu {\mathrm{H}}$)	$M_{t}^{{{r_2}}}$($\mu {\mathrm{H}}$)	$M_{t}^{{{r_3}}}$($\mu {\mathrm{H}}$)	$M_{{{r_1}}}^{{{r_2}}}$($\mu {\mathrm{H}}$)	$M_{{{r_1}}}^{{{r_3}}}$($\mu {\mathrm{H}}$)	$M_{{{r_2}}}^{{{r_3}}}$($\mu {\mathrm{H}}$)
Value	17.44	5.66	6.72	−0.57	−0.08	−0.32

*Negative mutual inductance values indicate opposing magnetic flux orientations between the corresponding coil pairs under the given spatial arrangement.

The design-variable vector is defined as

$$x = [e_{t}^{{}} ,\,R_{t}^{{}} ,\,\,C_{t}^{{}} ,\,\,C_{{}}^{{r_{{\,1}} }} ,\,\,C_{{}}^{{r_{{\,2}} }} ,\,\,C_{{}}^{{r_{{\,3}} }} ]^{T} \,$$

where $e_{t}^{{}}$ is the transmitter excitation (input) voltage, $R_{t}^{{}}$ is the transmitter-side resistance parameter used in the equivalent circuit, $C_{t}^{{}}$ is the transmitter compensation capacitance, and $C_{{}}^{{{r_i}}}\,(i=1,\,2,\,3)$ are the receiver-side compensation capacitances associated with each receiver topology in Fig. 2. These variables are selected because they are practically adjustable at the design stage and strongly influence the delivered power distribution and system-level efficiency.

The objective is to maximize the total power transfer efficiency of the multi-receiver WPT system, defined as

$$\eta _{{total}}^{{}} \,(x)\,\, = \sum\limits_{{i = 1}}^{3} {\eta _{i}^{{}} \,(x)\,} = \sum\limits_{{i = 1}}^{3} {\frac{{P^{{r_{i} }} }}{{P_{t} }}}$$

where $P_{t}^{{}}$ is the transmitter input power and $P_{{}}^{{{r_i}}}$​​ is the power delivered to receiver *i*, both evaluated by the DT-based analysis module under steady-state conditions. To ensure practical operation, each receiver must satisfy its rated power requirement within an allowable tolerance. The receiver-specific constraints are expressed as

$$\left| {P^{{r_{1} }} - P_{o}^{{r_{1} }} } \right| = \varepsilon P_{o}^{{r_{1} }} ,\,\,\left| {P^{{r_{2} }} - P_{o}^{{r_{2} }} } \right| = \varepsilon P_{o}^{{r_{2} }} ,\,\,\left| {P^{{r_{3} }} - P_{o}^{{r_{3} }} } \right| = \varepsilon P_{o}^{{r_{3} }}$$

where $P_{{\mathrm{o}}}^{{{r_1}}}=12.5\,{\text{W and }}P_{{\mathrm{o}}}^{{{r_2}}}=P_{{\mathrm{o}}}^{{{r_3}}}=5\,{\text{W }}$ are the rated power requirement assigned in Sect. 3.1, and $\varepsilon =0.03$ denotes the operational tolerance margin $\varepsilon$. Within the proposed formulation, this efficiency metric is refined only after all receiver-specific feasibility constraints are satisfied, ensuring that performance improvement is pursued within the feasible operating region.

Accordingly, the constrained optimization problem is formulated as

1 $$\begin{gathered} \begin{array}{*{20}c} {Find} & {x = \{ e_{t} ,R_{t} ,C_{t} ,C^{{r_{i} }} \} } & {(i = 1,2,3)} \\ {to\,\,\max imize} & {f = \eta _{{total}} (x)} & {\left( \% \right)} \\ {subject\,to} & {g_{i} = 1 - \frac{{P_{{}}^{{r_{i} }} \,(x)}}{{(1 - \varepsilon )P_{o}^{{r_{i} }} }} \le 0} & {\left( W \right)} \\ \end{array} \hfill \\ \begin{array}{*{20}c} {\,\,\,\,\,\,\,\,\,\,\,\,\,\,\,\,\,\,\,\,\,\,\,\,\,\,\,\,\,\,} & {g_{{i + 1}} = \frac{{P_{{}}^{{r_{i} }} \,(x)}}{{(1 + \varepsilon )P_{o}^{{r_{i} }} }} - 1\,\, \le \,\,0} & {\left( W \right)} \\ \end{array} \hfill \\ \begin{array}{*{20}c} {where} & {1 \le e_{t} \le 100} & {\left( V \right)} \\ {\,\,\,\,\,\,\,\,\,\,\,\,\,\,\,\,\,\,\,\,\,\,\,\,\,\,\,\,\,\,\,\,\,\,\,\,\,\,\,\,\,\,\,} & {0.1 \le R_{t} \le 10} & {\left( \Omega \right)} \\ {\,\,\,\,\,\,\,\,\,\,\,\,\,\,\,\,\,\,\,\,\,\,\,\,\,\,\,\,\,\,\,\,\,\,\,\,\,\,\,\,\,\,\,\,} & {0.1 \le C_{t} \le 100} & {\left( {nF} \right)} \\ {\,\,\,\,\,\,\,\,\,\,\,\,\,\,\,\,\,\,\,\,\,\,\,\,\,\,\,\,\,\,\,\,\,\,\,\,\,\,\,\,\,\,\,\,\,\,\,\,\,} & {0.1 \le C^{{r_{i} }} \le 100} & {nF} \\ \end{array} \hfill \\ \end{gathered}$$

where $P_{{}}^{{{r_i}}}$ is the power delivered to receiver *i* and the lower and upper bound of each design variable (​${\mathbf{x}}$) denote the design-variable bounds selected for practical tunability and stable circuit operation (reported together with the optimization formulation). This formulation directly reflects the system-level requirement that heterogeneous receivers must be simultaneously supported under a single transmitter while maintaining feasible and regulated power delivery across all receiver modules.

### Constraint-consistent operating-point convergence

**Fig. 4:**
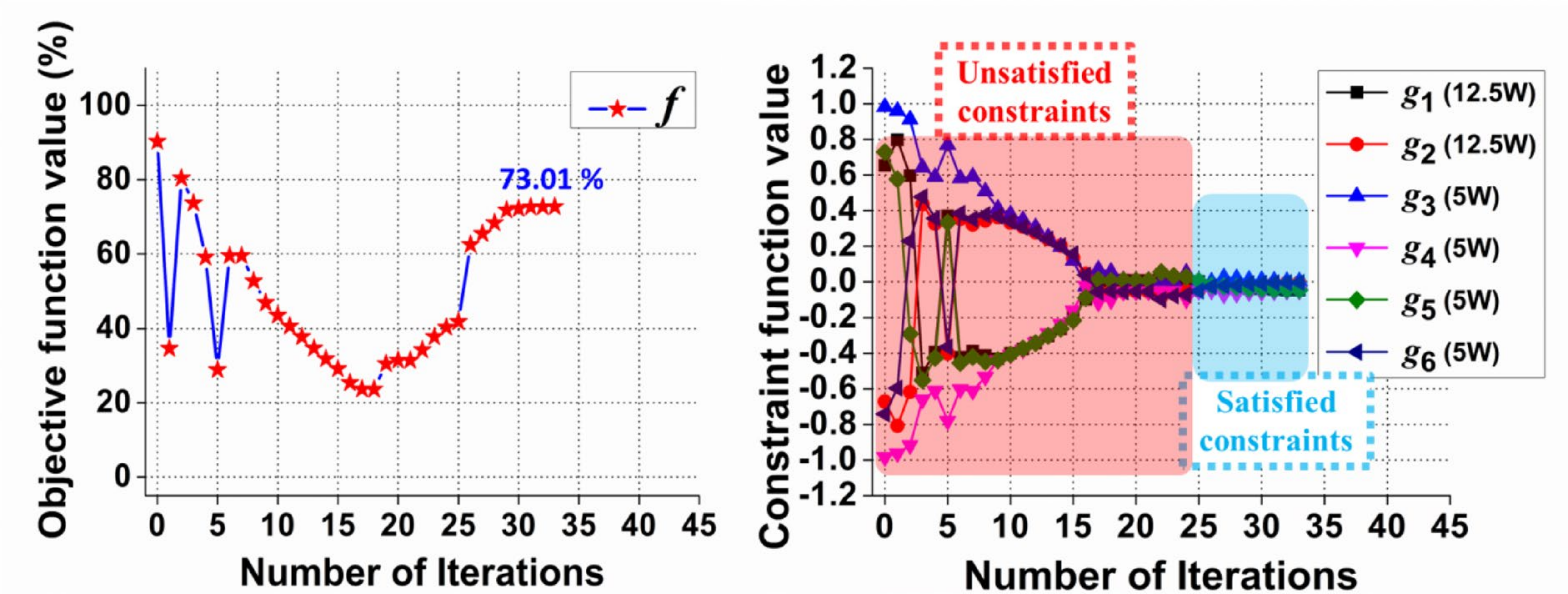
Convergence behavior of the constraint-consistent operating-point determination process: evolution of total power transfer efficiency (left) and receiver-specific feasibility constraints (right). A zero value indicates that the corresponding constraint is active (i.e., exactly satisfied).

Figure 4 summarizes the convergence behavior of the constraint-consistent operating-point determination process in terms of the objective function and receiver-specific constraint functions. The left-hand plot shows the evolution of total power transfer efficiency, while the right-hand plot reports the constraint-function values defined in Eq. (1). An operating point is considered feasible when the inequality conditions in Eq. (1) are satisfied within the prescribed tolerance.

During the initial iterations, the procedure prioritizes feasibility and searches for an operating region in which all receiver power constraints are satisfied. In this phase, the efficiency may exhibit non-monotonic variation as constraint violations are reduced. This reflects the feasibility-first nature of the operating-point determination process.

Once the receiver power constraints enter the feasible region, the operating condition is refined within the feasible domain. In this stage, the design variables are adjusted to improve total transmission efficiency while maintaining compliance with receiver-specific requirements. As shown in Fig. 4, this transition is characterized by stabilization of constraint values near zero and a gradual improvement in efficiency.

**Fig. 5:**
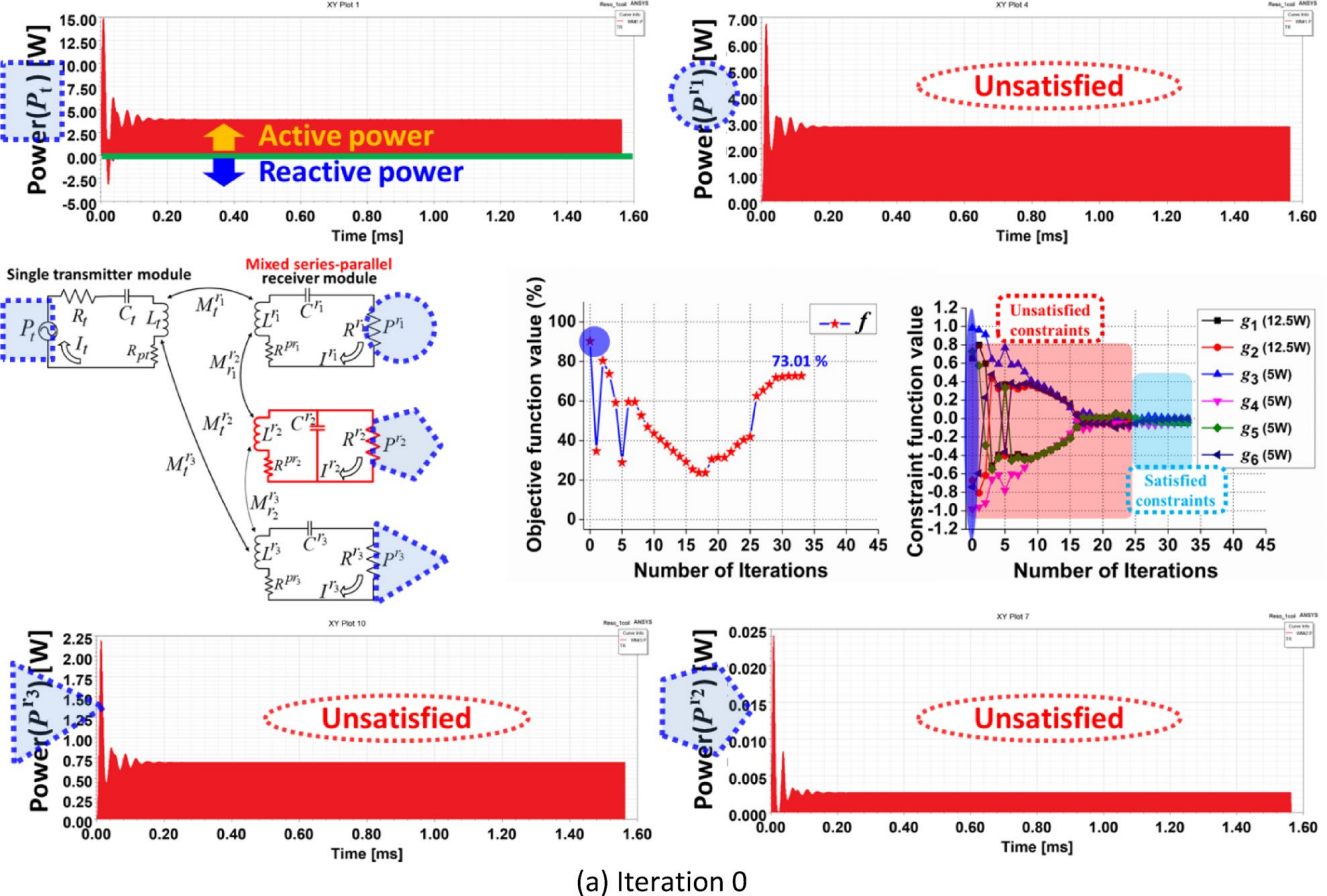

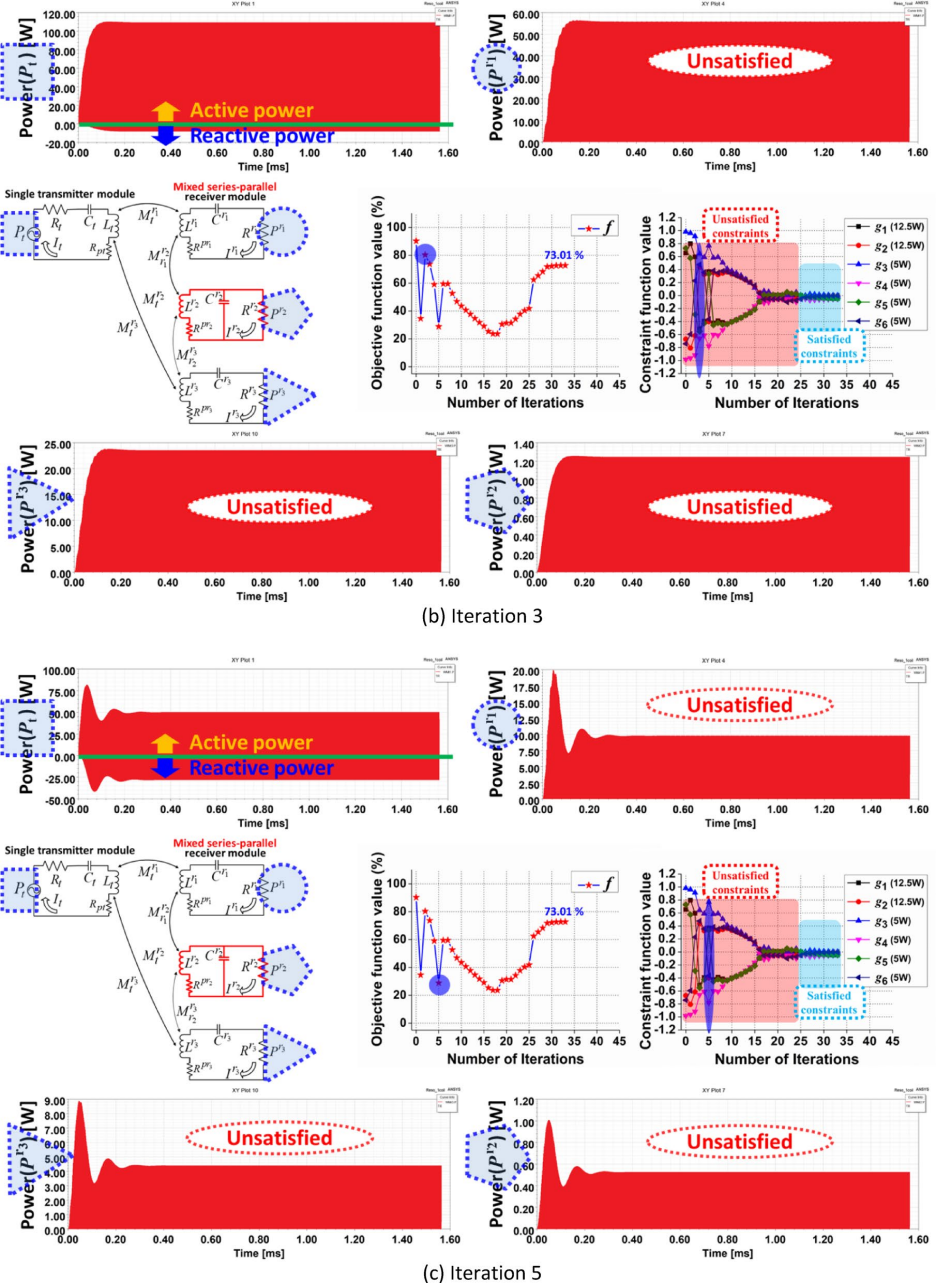

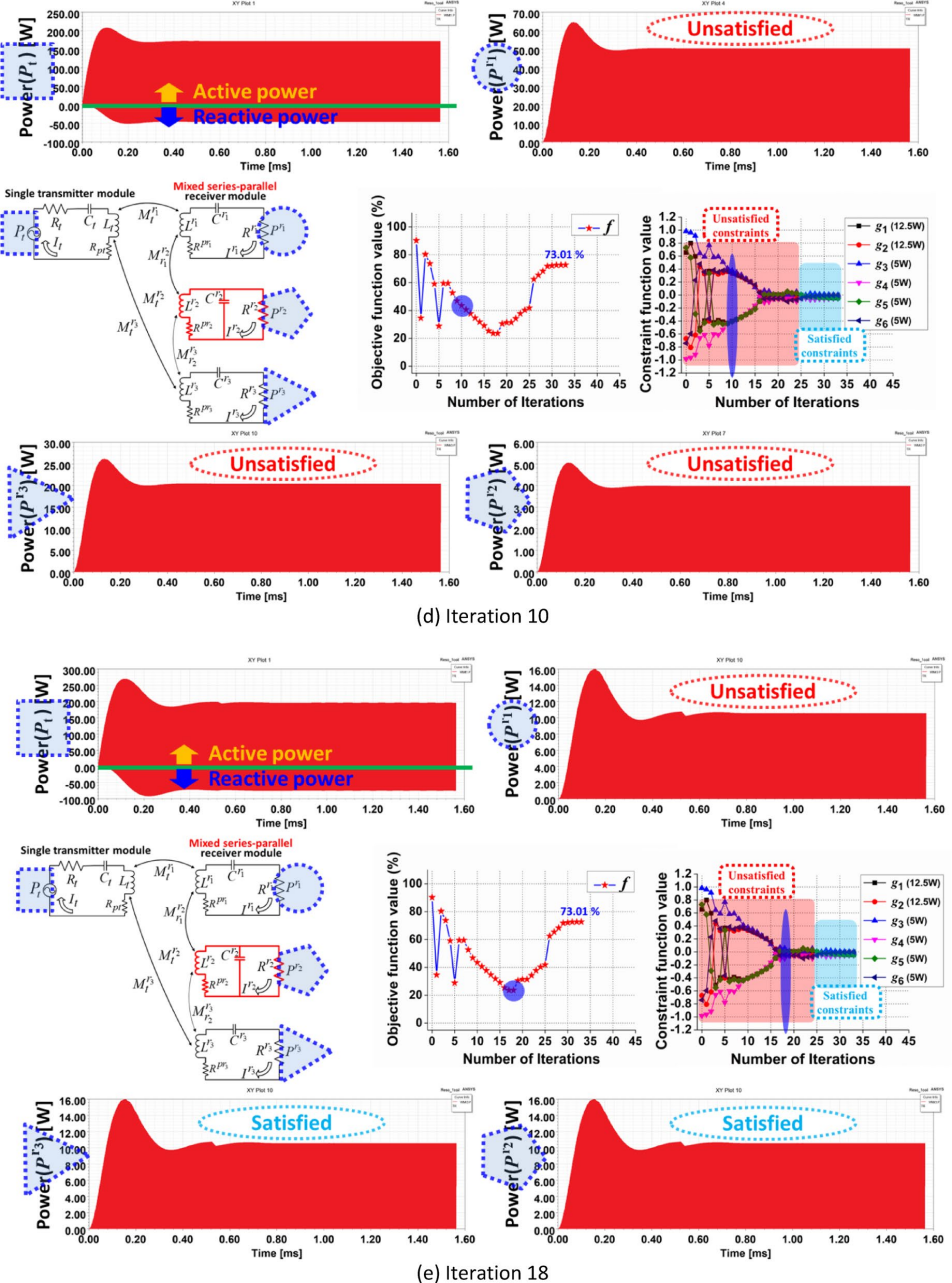

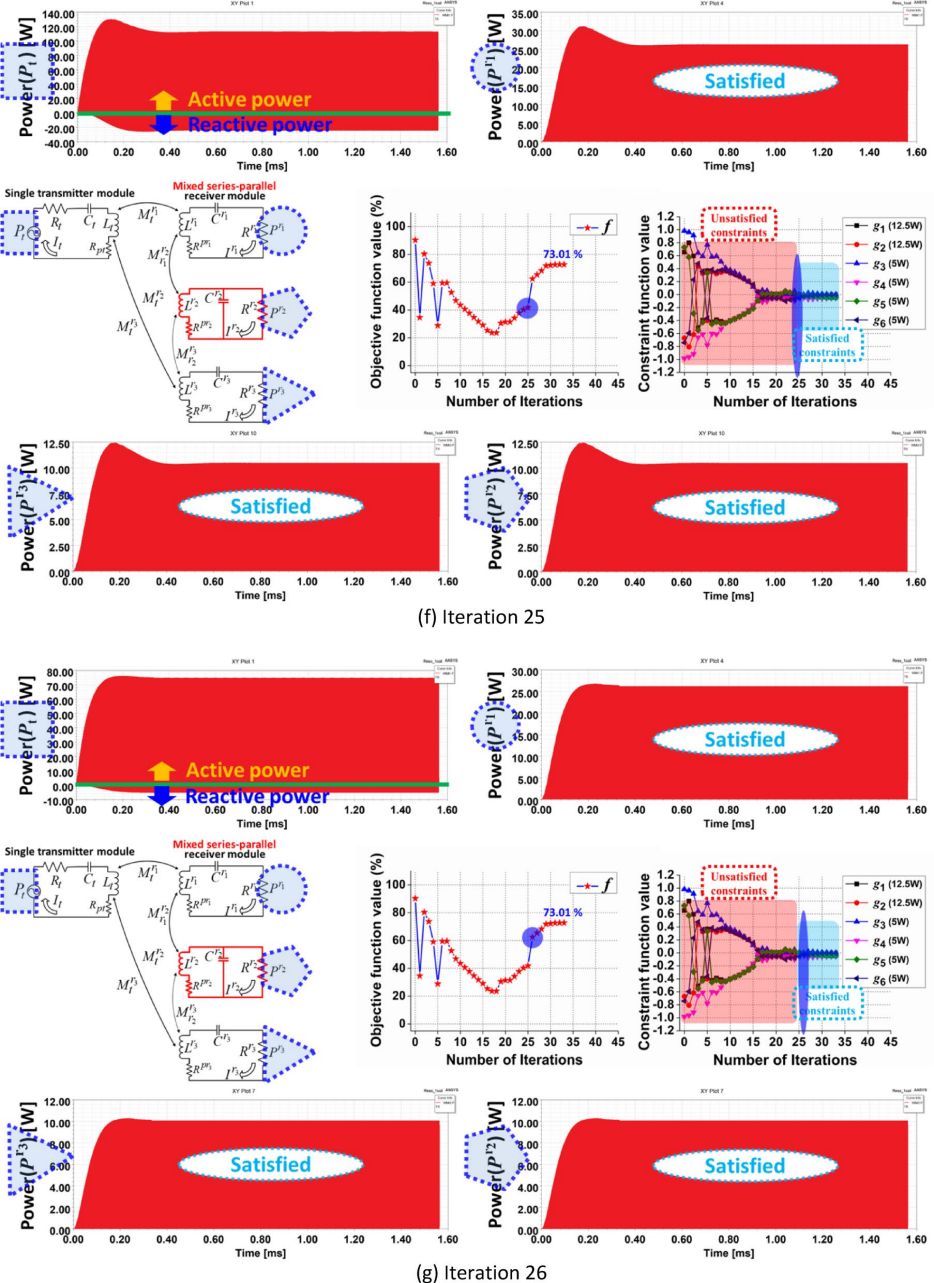

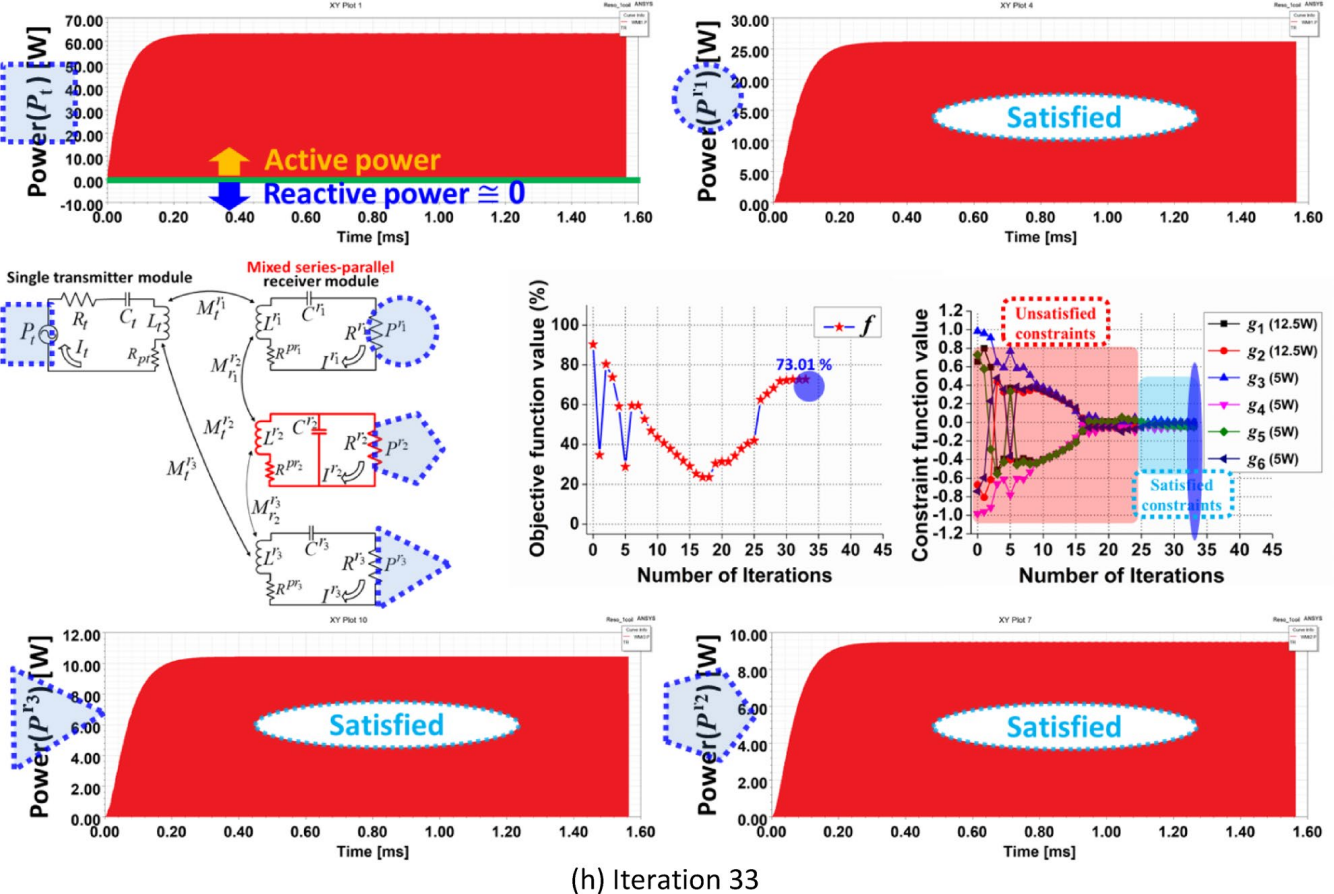
Iterative evolution of delivered power and reactive power during constraint-consistent operating-point determination in the 1T–3R WPT system.

To provide a physical interpretation of the convergence process, Fig. 5 illustrates representative iterations in terms of delivered power and reactive power. Early iterations may yield operating points with relatively high efficiency but unsatisfied receiver constraints, indicating infeasible operation despite favorable resonance indicators. Subsequent iterations enforce feasibility and then refine performance within the feasible region. The converged operating condition satisfies all receiver constraints and exhibits resonance-consistent behavior, as indicated by near-zero reactive power.

Convergence is declared when changes in the objective function become sufficiently small and all receiver power constraints remain satisfied within tolerance. Overall, Figs. 4 and 5 demonstrate that the proposed digital twin modelling approach systematically transitions from infeasible to feasible operation and identifies a constraint-consistent optimal operating condition for heterogeneous multi-receiver configurations.

### Constraint-consistent operating condition and system performance

This subsection presents the system performance evaluated at the converged constraint-consistent operating condition, with emphasis on receiver-level feasibility and system-level power transfer characteristics. While the convergence pathway is described in Sect. 3.3, the focus here is on the physical and operational implications of the identified operating point.

Table 3 summarizes the design variables and the resulting receiver-level delivered power and total transmission efficiency at convergence. The delivered powers match the rated power requirements of all receivers within the prescribed tolerance margins. In particular, the higher-power receiver (*r*_1_​) satisfies its rated demand, while the lower-power receivers (*r*_2_​ and *r*_3_​) also meet their requirements despite employing different compensation topologies. These results confirm that the digital twin–based modelling approach successfully enforces receiver-specific feasibility constraints in a heterogeneous multi-receiver configuration under a single transmitter.

The system-level transition can be interpreted by comparing the initial and converged operating conditions shown in Fig. 5. At Iteration 0, the system exhibits relatively high efficiency but violates receiver power constraints, demonstrating that efficiency alone does not guarantee feasible operation in heterogeneous multi-receiver systems. At the converged iteration (Iteration 33), all receiver constraints are satisfied (Table 3), and the reactive power approaches near-zero values, indicating resonance-consistent behavior under the imposed constraints. This comparison highlights the central outcome of the proposed approach: it identifies a constraint-consistent operating condition and subsequently refines performance within the feasible region.

For completeness, the computational cost associated with the design-stage iterative procedure is summarized in Table 4. This information characterizes the offline modelling effort required to determine the constraint-consistent operating condition.

**Table 3:** Constraint-consistent operating variables and receiver-level power delivery at convergence for the 1T–3R WPT system.

Category		Value
Operating variables at convergence	${e_t}$(*V*)	33.75
	${R_t}$(Ω)	1
	${C_t}$(*nF*)	0.44
	$C_{{}}^{{{r_1}}}$(*nF*)	1.94
	$C_{{}}^{{{r_2}}}$(*nF*)	3.67
	$C_{{}}^{{{r_3}}}$(*nF*)	4.67
Receiver-level performance	$P_{{}}^{{{r_1}}}$(*W*)	12.05
	$P_{{}}^{{{r_2}}}$ (*W*)	4.98
	$P_{{}}^{{{r_3}}}$ (*W*)	5.05
Total transmission efficiency	$\eta$(*%*)	73.01

**Table 4:** Computational cost associated with the design-stage operating-point determination procedure in the 1T–3R WPT system.

Category	Value
Number of iterations	33
Total number of circuit evaluations	543
Average circuit evaluation time (min)	0.5
Total runtime (hr)	4.52

*Electromagnetic-field simulation was performed once for parameter extraction; subsequent evaluations correspond to circuit-level simulations.

### Validation via one-dimensional (1D) parameter sweep

This subsection validates the local consistency and robustness of the constraint-consistent operating condition identified in Sect. 3.3–3.4 through a one-dimensional (1D) parameter sweep. Rather than introducing additional search iterations, this analysis examines whether the converged operating condition represents a locally feasible and performance-consistent solution within the surrounding design space.

For this purpose, each operating variable (i.e., *e*_*t*_, *C*_*t*_, *R*_*t*_, and *C* ^*ri*^ ​) was individually perturbed around the converged values summarized in Table 3, while all other variables were held fixed. For each perturbed configuration, the digital twin analysis module recomputed the total transmission efficiency and the receiver-specific delivered power values.

**Fig. 6:**
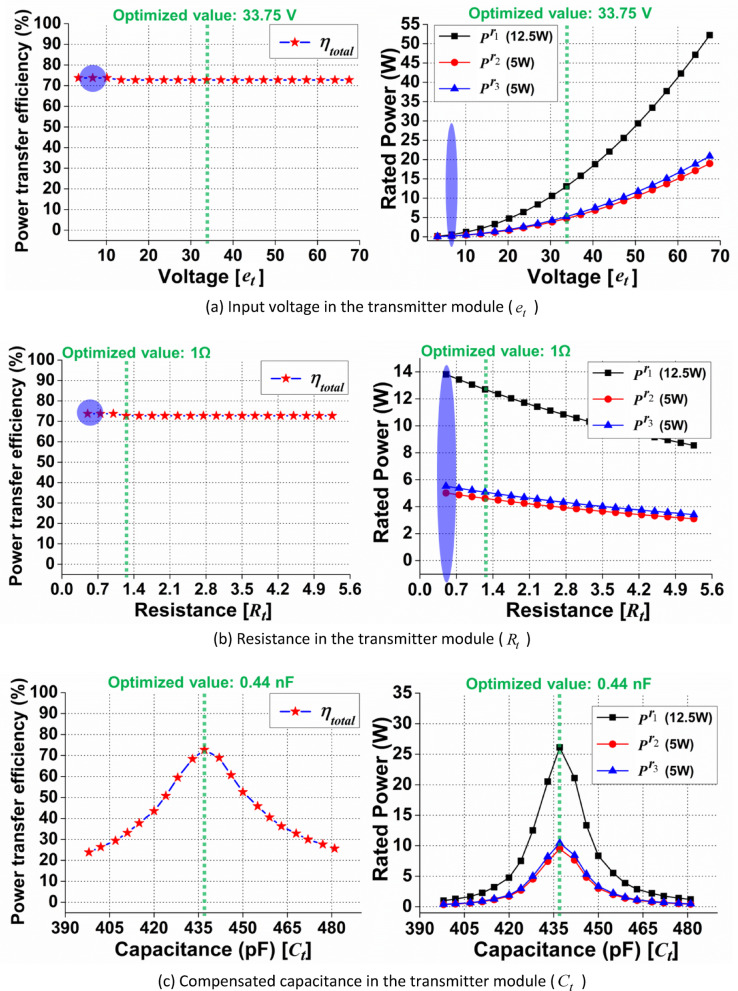

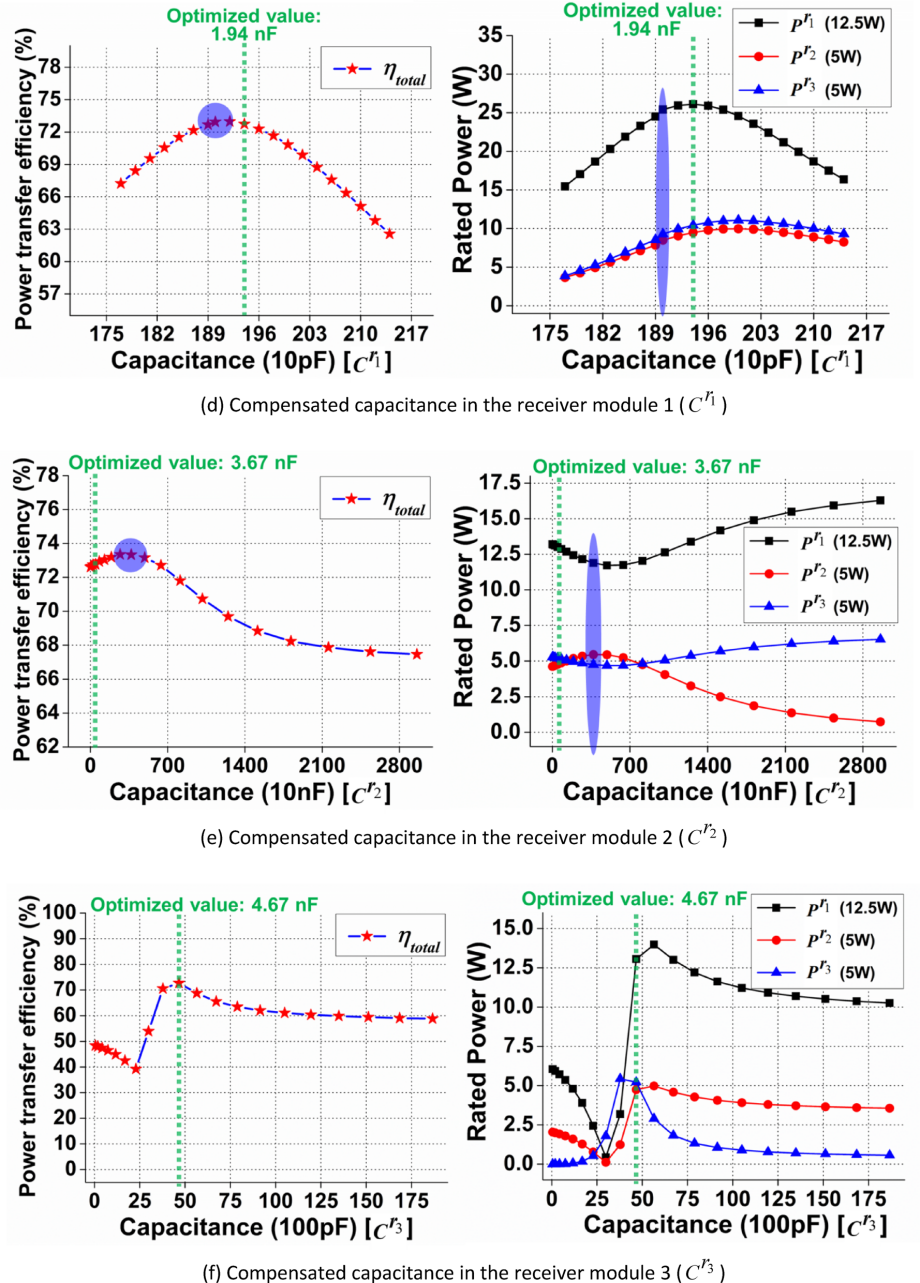
One-dimensional (1D) parameter sweep illustrating the local sensitivity of total power transfer efficiency and receiver-level delivered power around the constraint-consistent operating condition for each design variable.

This procedure enables direct evaluation of local sensitivity and reveals how system performance varies in the vicinity of the identified operating condition. As shown in Fig. 6, the total transmission efficiency generally reaches its maximum in the neighborhood of the converged operating point, while receiver delivered power approaches the prescribed rated values. These observations confirm that the identified operating condition satisfies receiver-specific constraints and lies within a locally performance-consistent region of the design space. In several subplots, specifically in Figs. 6a,b,d,e, the point of maximum efficiency does not coincide exactly with the point where all constraints are simultaneously satisfied. This reflects the intrinsic trade-off between maximizing efficiency and enforcing receiver-specific feasibility requirements. The proposed procedure therefore prioritizes feasibility and subsequently refines efficiency within the feasible region, ensuring that the resulting operating condition remains physically consistent.

Beyond feasibility verification, the 1D sweep provides insight into the relative influence of individual variables on system behavior. The observed sensitivity patterns indicate which parameters most strongly affect performance and suggest directions for future investigations, such as tolerance-aware modelling or adaptive adjustment strategies.

Overall, the 1D parameter sweep supports the validity of the identified constraint-consistent optimal operating condition and demonstrates the capability of the digital twin modelling approach to analyze heterogeneous multi-receiver WPT systems in a reproducible and physics-consistent manner.

## Discussion

The results in Sect. 3 show that heterogeneous multi-receiver WPT systems should be interpreted as constrained multi-load operating problems rather than as collections of independently tuned resonant links. In this context, the primary outcome of this study is not merely a higher transmission efficiency, but the ability to determine an operating condition that simultaneously satisfies receiver-specific power requirements under a single transmitter despite mixed compensation topologies and non-uniform rated demands. A key novelty is that the proposed electromagnetic–circuit digital twin enables the explicit selection of a constraint-consistent optimal operating condition, distinguishing feasible operation from resonance- or efficiency-favored but constraint-violating points. This provides a reproducible and physics-consistent pathway for identifying the optimal operating point within the feasible region for heterogeneous multi-receiver configurations.

A central observation arising from the convergence behaviour is that high transmission efficiency alone does not guarantee feasible operation in multi-receiver systems. Operating points that appear well tuned from a resonance standpoint may still violate receiver-level power constraints, especially when receiver requirements are heterogeneous and coupling interactions are strong. By explicitly formulating receiver power requirements as constraints and evaluating them within the design-stage loop, the proposed approach systematically navigates the trade-off between feasibility and performance: it first reaches the feasible domain and then refines performance within that domain. The one-dimensional parameter sweep further supports the local validity of the obtained operating point by showing that the converged solution lies near a locally favourable region while satisfying all receiver constraints, and it highlights sensitivities that may inform tolerance-aware design or adaptive strategies in future studies.

Several limitations of the present study should be noted. The case study assumes a fixed physical layout and a predefined number of receivers, and electromagnetic coupling parameters are treated as constants once extracted. These assumptions limit the direct applicability of the framework to practical WPT environments involving positional variation, time-varying loads, and changing receiver configurations. In particular, battery-powered loads exhibit stage-dependent behavior, such as constant-current and constant-voltage operation, which introduces time-varying effective load conditions. Addressing such effects requires representing receiver-side loads as time-dependent parameters or discrete charging states and evaluating the feasible operating region across these conditions. In addition, positional shift or misalignment modifies the mutual inductance and coupling coefficients, which can alter both the feasible operating region and the resulting optimal operating condition. Addressing these effects requires extending the present fixed-parameter digital twin to a position-dependent or state-dependent formulation, in which the mutual inductance matrix is updated across representative spatial configurations or through an online update mechanism.

A further extension is required for practical scenarios involving receiver connection and disconnection. In such cases, the effective system topology and the associated constraint interactions change with the active receiver set. Incorporating these effects requires a reconfigurable receiver-state representation, allowing the operating-point determination process to adapt to changing receiver configurations. Taken together, these extensions transform the current fixed-parameter digital twin into a state-dependent or updateable framework, supporting robust operating-point determination under dynamically varying WPT conditions.

The present results demonstrate that integrating electromagnetic modelling, circuit-level analysis, and constraint-aware operating-point determination within a digital twin provides a practical basis for analyzing and designing heterogeneous multi-receiver WPT systems. However, as the number of receivers increases, the dimensionality of the coupling matrix and the number of interacting constraints increase significantly, leading to stronger nonlinear coupling and increased complexity of the operating-point determination problem. In the present framework, electromagnetic parameters are extracted once for a fixed configuration, and the dominant computational cost arises from repeated circuit-level evaluations. Therefore, scalability challenges are primarily associated with increased circuit-model dimensionality and convergence complexity of the constrained search. Addressing these challenges requires methodological extensions such as decomposition strategies, reduced-order modelling, or more scalable optimization approaches.

## Conclusion

This study addressed a central challenge in heterogeneous multi-receiver wireless power transfer (WPT) systems: determining an operating condition that is simultaneously feasible for all receivers and efficient at the system level under strong electromagnetic coupling and mixed compensation topologies. We presented an integrated electromagnetic–circuit digital twin modelling approach that enables constraint-consistent optimal operation, by explicitly distinguishing feasible operating points from resonance- or efficiency-favored but constraint-violating points and then refining performance within the feasible region. The approach was demonstrated on a representative single-transmitter, three-receiver (1T–3R) system comprising mixed series- and parallel-compensated receivers with non-uniform rated power requirements. The results showed a clear transition from infeasible to feasible operation and subsequent performance refinement, while satisfying receiver-specific power constraints. A one-dimensional parameter sweep around the obtained operating point further supported local robustness and sensitivity characteristics, providing additional confidence in the validity of the constraint-consistent optimum.

The present study considers a fixed physical layout and a predefined receiver set, with electromagnetic coupling parameters extracted for the given configuration. Extension of the proposed approach to dynamic scenarios involving misalignment and time-varying loads requires additional modelling layers and updating of coupling conditions. In addition, the current validation is limited to a simulation-based digital-twin environment, and experimental verification using a physically constructed prototype is not included in this study. Therefore, future work will focus on prototype implementation and experimental validation of the proposed framework, including comparison between digital-twin predictions and measured system performance under practical operating conditions. Nevertheless, the proposed digital twin modelling provides a reproducible and physics-consistent pathway for analyzing and designing heterogeneous multi-receiver WPT systems, and serves as a foundation for future studies of more complex and dynamically varying wireless power transfer configurations.

## Supplementary Information

Below is the link to the electronic supplementary material.

Supplementary Material 1

Supplementary Material 2

## Data Availability

The datasets generated and analyzed during the current study are available from the corresponding author upon reasonable request.
